# Resolution Comparison of a Standoff Gel Pad Versus a Liquid Gel Barrier for Nasal Bone Fracture Sonography: A Standardized Crossover Study

**DOI:** 10.3390/diagnostics16010092

**Published:** 2025-12-26

**Authors:** Dong Gyu Kim, Kyung Ah Lee

**Affiliations:** Department of Plastic and Reconstructive Surgery, Inje University Haeundae Paik Hospital, Busan 48108, Republic of Korea

**Keywords:** nasal bone fracture, ultrasonography, contrast-to-noise ratio, signal-to-noise ratios

## Abstract

**Background**: High-frequency ultrasonography (US) is increasingly used to guide closed reduction in nasal bone fractures, but near-field resolution over the curved nasal dorsum depends critically on the acoustic coupling medium. We aimed to determine whether a semi-solid standoff gel pad (PAD) provides superior image contrast and signal stability compared with a liquid gel barrier (LGB) during intraoperative nasal bone fracture sonography. **Methods**: In this prospective, single-center, within-subject crossover study, 30 adults with isolated nasal bone fractures underwent intraoperative high-frequency US of the nasal dorsum under two coupling conditions differing only by the medium used: a 7 mm hydrogel standoff pad (PAD) and a custom-made 7 mm liquid gel barrier (LGB). All scans were acquired on the same platform using fixed B-mode presets (10 MHz, 4.0 cm depth, single focal zone at the cortex). Rectangular regions of interest (ROIs) were placed on the cortical interface (bone ROI) and adjacent soft tissue (soft-tissue ROI) at matched depth. For each subject and condition, contrast-to-noise ratio (CNR) and two signal-to-noise ratios (SNR_bone, SNR_soft) were derived from ROI gray-level statistics and compared using paired *t*-tests. **Results**: The PAD yielded a significantly higher CNR at the cortical interface compared to the LGB (3.46 ± 0.17 vs. 2.50 ± 0.19; mean paired difference 0.96, 95% CI 0.88–1.04; *p* < 0.0001). SNR_bone was also higher with PAD (4.31 ± 0.35 vs. 3.63 ± 0.34; difference 0.68, 95% CI 0.52–0.83; *p* < 0.0001). Using the soft-tissue ROI as the noise reference (SNR_soft), PAD again outperformed LGB (7.64 ± 0.73 vs. 6.68 ± 0.78; difference 0.96, 95% CI 0.59–1.33; *p* = 0.000012). **Conclusions**: Compared with a liquid gel barrier of similar thickness, a semi-solid standoff gel pad provides higher near-field CNR and SNR at the nasal cortical interface under standardized intraoperative conditions. These quantitative differences support the use of a gel pad as a practical coupling medium for real-time ultrasound guidance during closed reduction in nasal bone fractures, although the impact on clinical outcomes remains to be determined.

## 1. Introduction

Nasal bone fracture is the most common facial fracture. Closed reduction benefits from real-time visualization of cortical discontinuities and step-offs. High-resolution ultrasonography (US) provides such guidance without ionizing radiation and shows diagnostic utility in both pediatric and adult populations [1,2,3,4,5].

On the curved nasal dorsum, near-field performance depends on the acoustic medium at the probe–skin interface. Micro-air interfaces and unstable contact diminish contrast and obscure thin cortical lines. Water-bath coupling can provide wide-field views but is vulnerable to bubbles, meniscus curvature, and motion during manipulation, limiting intraoperative practicality [6].

From an acoustic standpoint, soft-tissue–air boundaries reflect most incident energy; uninterrupted, air-free coupling is therefore essential. Bench studies favor gel-based media for transmissivity and impedance matching compared with water or oils [7,8,9]. For superficial targets, standoff pads extend the effective near field and stabilize contact; clinical reports describe improved Doppler detectability and preserved elastography performance with pad interposition [10,11].

We aimed to determine whether a semi-solid standoff gel pad (PAD) confers superior resolution relative to a liquid gel barrier (LGB) in nasal bone fracture sonography, using a standardized acquisition protocol and an objective image-based contrast surrogate under fixed presets [12,13,14].

## 2. Materials and Methods

### 2.1. Study Objective and Design

This prospective, single-center, within-subject standardized crossover study was designed to evaluate whether the choice of acoustic coupling medium affects image resolution in nasal bone fracture sonography. This study was conducted in accordance with the Declaration of Helsinki and was approved by the Institutional Review Board (IRB) of Inje University Haeundae Paik Hospital (IRB No. 2023-10-022-002; approval date 15 December 2023). Each patient with an isolated nasal bone fracture underwent US of the nasal dorsum under two conditions that differed only by the coupling medium: PAD and LGB. The primary objective was to compare the contrast-to-noise ratio (CNR) at the cortical interface between PAD and LGB during intraoperative sonography for closed reduction. Secondary objectives were to compare signal-to-noise ratios (SNR) of the cortical and adjacent soft-tissue regions of interest (ROI) under the two media.

### 2.2. Participants

Adults (≥18 years) with isolated nasal bone fractures scheduled for closed reduction were eligible. Exclusion criteria were: (i) concomitant facial fractures, (ii) open wounds or skin conditions precluding safe application of coupling media over the nasal dorsum, (iii) prior nasal surgery within 6 months, and (iv) inability to cooperate with the intraoperative US protocol (e.g., inability to maintain supine position). Thirty consecutive patients who satisfied these criteria and completed imaging under both coupling conditions were included in the analysis.

### 2.3. Order of Conditions and Masking

To standardize acquisition and reduce variability related to repositioning and residual gel, all participants were scanned in a fixed sequence: the PAD condition was always acquired first, followed by the LGB condition. After completion of the PAD sweep, the nasal skin and surrounding perinasal area were thoroughly wiped and allowed to dry before the LGB assembly was positioned, in order to minimize bias of residual gel between conditions.

Before quantitative analysis, all exported images were anonymized. A single reader performed all ROI placement and intensity measurements. To assess reliability, this reader repeated all measurements in a random subsample comprising 20% of the PAD/LGB image pairs, and agreement between the repeated CNR, SNR_bone, and SNR_soft values was quantified as described in Section 2.8.

### 2.4. Ultrasound System and Acquisition Presets

All examinations were performed using a S3 ultrasound system (ZONARE Medical Systems, Inc., Mountain View, CA, USA) equipped with a linear L14-5w transducer (operating range 5–14 MHz; 55 mm field of view). Imaging was carried out in 2D B-mode only. Acquisition parameters were held constant for both PAD and LGB conditions: depth 4.0 cm, transmit frequency 10 MHz, single focal zone positioned at the cortical interface, dynamic range 75 dB, overall gain 76, and tissue harmonic, compound imaging, persistence, and other post-processing filters switched off. These fixed presets were chosen to emphasize superficial cortical detail. Images were exported as DICOM or uncompressed PNG in 8-bit (0–255) without additional window/level adjustment.

### 2.5. Coupling Media

Standoff gel pad (PAD): A hydrogel standoff pad (thickness 7 mm, diameter approximately 90 mm; SOLID GEL PAD^®^, Bluemtech, Republic of Korea) was centered over the nasal dorsum, with a thin smear of standard ultrasound gel (ECO GEL 99^®^, Seungwon Medical Co., Ltd., Bucheon, Republic of Korea) applied only to wet the skin–pad interfaces. The pad thickness was selected to place the nasal cortex within the proximal near field of the 14 MHz transducer while providing a stable, conformal contact surface.

**Figure 1:**
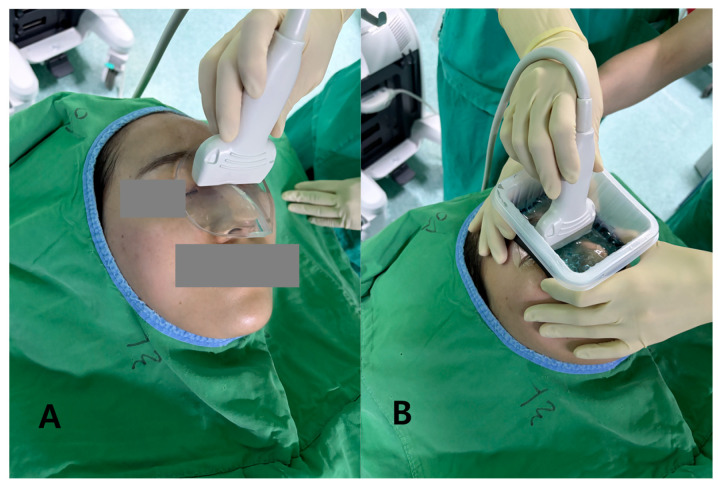
Intraoperative photographs showing how the two coupling media were used during nasal bone fracture sonography. (**A**) 7 mm hydrogel standoff pad (PAD) is placed over the nasal dorsum and the linear probe is positioned directly on top of the pad with minimal pressure. (**B**) The same probe is applied over a liquid gel barrier (LGB). In this setup, a shallow perinasal plastic container with an internal waterproof sponge liner is secured around the nose and filled with sterile ultrasound gel.

Liquid gel barrier (LGB): To create an LGB, we assembled a shallow perinasal plastic container by affixing a narrow band of waterproof closed-cell sponge to the inner rim of a circular plastic ring. This assembly was secured to the perinasal skin using a transparent adhesive drape, forming a low-profile, sealed reservoir that confined the gel around the nasal dorsum. Approximately 10 mL of ultrasound gel was injected through a luer-lock port while a small air vent ensured evacuation of bubbles, targeting a gel layer thickness up to 7 mm. The LGB was designed to mimic a liquid near-field standoff while matching the PAD in effective depth and footprint so that differences in image quality predominantly reflected the medium state (semi-solid vs. liquid) (Figure 1).

During filling, the gel surface was inspected visually and the container was gently tapped to dislodge residual air bubbles; if focal accumulations of bubbles or obvious meniscus irregularities were seen along the beam path, the gel was aspirated and refilled before imaging. These steps were intended to reduce artifacts related to trapped air and uneven gel thickness.

### 2.6. Image Acquisition Protocol and Handling

With the patient in the supine position and the head slightly elevated, the transducer was oriented longitudinally along the nasal dorsum. For each condition, an approximately 3 s linear sweep was recorded over the central dorsum without changing presets. The same operator performed all acquisitions and was instructed to apply the minimum pressure required to maintain consistent contact, thereby minimizing probe-induced deformation of the nasal framework. Before each sweep, the operator confirmed in real time that the near field and the nasal cortex were not obscured by obvious reverberation, streak artifacts, or bubble-related clutter at the gel–skin or gel–pad interfaces; if such artifacts were observed, probe position and coupling were adjusted and a new sweep was acquired. After completion of the PAD sequence, the perinasal skin was cleaned, the LGB was applied as described above, and the LGB acquisition was performed with identical probe orientation and sweep path.

### 2.7. ROI Placement for CNR/SNR Measurements

All ROI-based analyses were performed using Fiji (ImageJ; National Institutes of Health, Bethesda, MD, USA), v1.52. For each subject and condition, one representative PAD image and one representative LGB image were selected from the recorded cine loops, prioritizing frames in which the cortical surface was sharp and free of motion, reverberation artifacts, or obvious bubble-related clutter in the near field.

**Figure 2:**
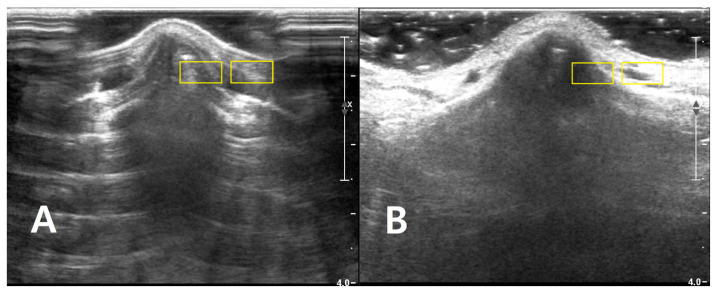
Longitudinal B-mode ultrasound images of the nasal dorsum in a volunteer illustrating ROI placement for quantitative analysis of each (**A**) PAD and (**B**) LGB image. Two rectangular ROIs (approximately 6 × 3 mm, yellow boxes) are drawn at the same depth. The image of cortical ROI was confined to the bright nasal cortical line, avoiding saturated pixels and obvious artifacts, and the adjacent soft-tissue ROI is positioned 1–2 mm lateral at the same depth to sample homogeneous soft tissue for CNR and SNR calculations. The images are provided for illustrative purposes to demonstrate ROI placement and were acquired.

**Figure 3:**
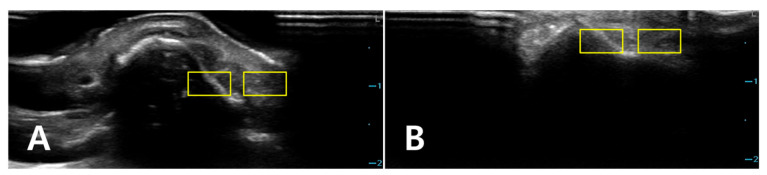
Representative sonograms of isolated nasal bone fractures demonstrating ROI placement under two coupling conditions. For two different patients, longitudinal B-mode images of the nasal dorsum are shown with (**A**) PAD and (**B**) LGB. Two rectangular ROIs (approximately 6 × 3 mm; yellow boxes) were positioned at a matched depth in each image. One ROI was placed along the hyperechoic nasal cortical line, with care taken to avoid saturated pixels and obvious artifacts. A second, adjacent ROI was positioned 1–2 mm lateral at the same depth to sample homogeneous soft tissue. These examples illustrate the standardized ROI placement used across PAD and LGB images for subsequent CNR and SNR analyses. All images were acquired using the ultrasound presets described in Section 2.4 (depth, 4.0 cm; single focal zone at the nasal cortical interface). The images were cropped by 2.0 cm in the vertical direction to exclude the non-informative anechoic coupling medium visible on the screen; no additional rescaling was performed.

Two rectangular ROIs were defined at a fixed depth and orientation on the PAD image and then reapplied to the LGB image with only minor adjustments to maintain alignment: Cortical ROI (ROI_bone): A small rectangle (approximately 6 × 3 mm) confined to the bright cortical band at the nasal dorsum, avoiding saturated pixels (intensity = 255) and excluding overlying reverberation or shadowing. Adjacent soft-tissue ROI (ROI_soft): A rectangle of similar size placed 1–2 mm lateral to the cortical ROI at the same depth, sampling homogeneous soft tissue (Figure 2). The exact lateral position of the ROIs was adjusted so that, in both PAD and LGB images, ROI_bone and ROI_soft remained aligned to the same anatomical level (depth mismatch ≤ 0.5 mm; angular mismatch ≤ 5°). For subjects with cine data, the same ROIs were propagated across five consecutive frames: the frame with the highest mean intensity within ROI_bone and its two preceding and two subsequent frames. For each frame, the mean (μ) and standard deviation (σ) of the 8-bit gray-level values were recorded for ROI_bone and ROI_soft. For subjects with only static images available, ROI_bone and ROI_soft were repositioned across three to five adjacent locations along the same cortical segment, and measurements were averaged (Figure 3).

### 2.8. Statistical Analysis

For each subject and each condition (PAD and LGB), the frame-level or spatial replicate measurements from the two ROIs were averaged so that one value per subject and condition was used in the analysis. ROI_bone provided the mean signal and its standard deviation for the nasal cortex, and the ROI_soft provided the corresponding background values.

The primary endpoint was the CNR at the cortical interface. CNR was defined at the subject level as the absolute difference between the mean signal in the ROI_bone and the mean signal in the ROI_soft, normalized by the combined noise level in these two regions. This metric reflects how strongly the cortex stands out from the adjacent soft tissue once the local noise is taken into account.

The secondary endpoint was the SNR of the cortical ROI (SNR_bone), calculated as the mean cortical intensity in ROI_bone divided by the standard deviation within ROI_bone.

The third endpoint was a soft tissue related signal-to-noise ratio, hereafter denoted SNR_soft, calculated by dividing the mean cortical intensity in ROI_bone by the standard deviation measured in the adjacent ROI_soft. This metric reflects the strength of the cortical signal relative to noise in the neighboring soft tissue.

For each of these metrics (CNR, SNR_bone and SNR_soft), PAD and LGB were compared using paired *t*-tests with a significance level of 0.05. For reporting, we present the mean ± standard deviation for each medium, the paired difference between PAD and LGB with a 95% confidence interval, and the corresponding t statistic, degrees of freedom and *p*-value.

For each metric (CNR, SNR_bone, and SNR_soft), an intraclass correlation coefficient (ICC) with a two-way mixed-effects model and absolute agreement definition was calculated, following published recommendations for reliability research [14].

## 3. Results

### 3.1. Image Set

Thirty paired PAD/LGB image sets from 30 noses were included in the analysis. All examinations were performed with the same preset (depth 4.0 cm, 10 MHz, dynamic range 75 dB, gain 76). PAD and LGB ROIs were placed at almost identical depths; the mean depth difference between the two media was 0.21 ± 0.10 mm.

### 3.2. Primary Endpoint: CNR at the Cortical Interface

At the nasal cortical interface, CNR was higher with the standoff gel pad than with the liquid gel barrier. The mean CNR was 3.46 ± 0.17 for PAD and 2.50 ± 0.19 for LGB. The mean paired difference (PAD − LGB) was 0.96 with a 95% confidence interval of 0.88 to 1.04. The paired *t*-test gave t(29) = 23.42, *p* < 0.0001. Under identical imaging settings, the cortical interface was therefore more clearly separated in contrast from the adjacent soft tissue when PAD was used.

### 3.3. Secondary Endpoints: SNR

The ROI_bone also showed higher SNR with PAD. SNR_bone was 4.31 ± 0.35 with PAD and 3.63 ± 0.34 with LGB, giving a mean paired difference of 0.68 (95% CI 0.52 to 0.83; t(29) = 9.05, *p* < 0.0001).

**Figure 4:**
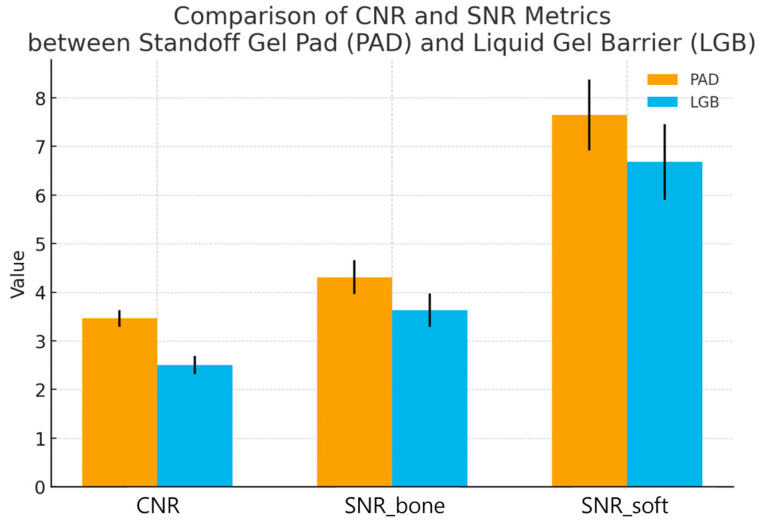
Quantitative comparison of image quality metrics between the standoff gel pad and the liquid gel barrier.

When the standard deviation in the adjacent ROI_soft was used as the noise reference (SNR_soft), the same trend was seen. SNR_soft was 7.64 ± 0.73 with PAD and 6.68 ± 0.78 with LGB. The mean paired difference was 0.96 (95% CI 0.59 to 1.33; t(29) = 5.26, *p* = 0.000012). These findings indicate that, in addition to higher contrast, the cortical signal was less affected by background noise when the standoff gel pad was used as the coupling medium (Figure 4).

### 3.4. Intraobserver Reproducibility

In the random subsample comprising 20% of the PAD/LGB image pairs (12 images), repeated ROI-based measurements by the same reader showed high intraobserver agreement. For CNR, the ICC was 0.97 (95% CI 0.91–0.99). For SNR_bone, the ICC was 0.93 (95% CI 0.78–0.98), and for SNR_soft, the ICC was 0.95 (95% CI 0.84–0.99), indicating excellent repeatability of the quantitative metrics under the present analysis protocol.

Paired quantitative results for 30 nasal bone fractures imaged with a semi-solid standoff gel pad (PAD) and a liquid gel barrier (LGB) under identical ultrasound presets. For each subject, contrast-to-noise ratio (CNR) at the nasal cortical interface and two signal-to-noise ratios (SNR_bone and SNR_soft) were calculated from matched cortical and adjacent soft-tissue ROIs. Bars indicate mean ± standard deviation, and individual subjects are shown as paired points connected by thin lines. CNR was higher with PAD than with LGB (3.46 ± 0.17 vs. 2.50 ± 0.19; *p* < 0.0001), indicating clearer separation of the cortex from adjacent soft tissue. The cortical SNR (SNR_bone) was also increased with PAD (4.31 ± 0.35 vs. 3.63 ± 0.34; *p* < 0.0001), and the soft tissue related SNR (SNR_soft) showed the same pattern (7.64 ± 0.73 vs. 6.68 ± 0.78; *p* = 0.000012). Together, these results show that the standoff gel pad provides a more stable and higher-contrast depiction of the nasal cortex than the liquid gel barrier in intraoperative nasal bone sonography.

## 4. Discussion

In this within-subject crossover study, the PAD consistently produced higher CNR and SNR at the nasal cortical interface than the LGB. This aligns with subjective visual assessment: when PAD is used, the cortical line and subtle step-offs tend to stand out more clearly from the surrounding soft tissue, and the near-field appears cleaner. High-frequency ultrasonography has already been shown to be useful for diagnosing and managing nasal bone fractures, but its performance over the nasal dorsum depends heavily on the coupling medium and on how well thin cortical structures can be distinguished from adjacent soft tissue [1,2,3,4,5]. Water-bath or immersion techniques can widen the field of view, but they are vulnerable to bubbles, meniscus changes and motion, and these factors often reduce near-field sharpness even when overall penetration is adequate [6].

Studies comparing acoustic media have reported that gel-based couplants provide better impedance matching and lower attenuation than water or oils, particularly for superficial targets [7,8]. Clinical and technical papers have also shown that gel standoff pads can improve near-field performance, for example, by enhancing Doppler signal detection or stabilizing shear-wave elastography of superficial lesions [9,10]. Our CNR and SNR findings aligned with these earlier observations. With PAD, the mean intensity difference between bone and adjacent soft tissue is larger relative to the combined noise, and the cortical signal itself is more stable relative to its own variability and to the variability in the neighboring soft tissue. Put simply, the cortex is more clearly separated from background fluctuations when a pad is used than when a liquid layer is retained by a barrier.

A plausible explanation is that the semi-solid pad provides a more stable and uniform near field. The pad conforms to the curved nasal dorsum and maintains a relatively constant, air-free path between the probe and the cortex, which reduces small reverberations and clutter close to the transducer. This should make the transition from soft tissue to bone sharper and more abrupt. By contrast, the LGB relies on a liquid layer confined by a plastic container and sponge liner, so any residual bubbles, local variations in gel thickness, or irregularities at the gel–air interface can introduce additional low-level echoes. These echoes can broaden the apparent transition between bone and soft tissue and lower local CNR, even if the overall brightness of the image looks acceptable.

Methodologically, we applied a fixed sequence in which PAD was always used first, followed by LGB. This approach was chosen to standardize the intraoperative workflow and to reduce variability arising from repeated repositioning and residual gel. It was designed to minimize practical sources of error compared with a randomized sequence in this setting.

From a practical standpoint, the LGB system used here was relatively complex, requiring assembly of a perinasal container and careful filling and venting of the gel. This design was chosen to approximate a liquid near-field standoff of similar thickness and footprint to the PAD under controlled conditions, but it may be less convenient for routine use and may be difficult to handle in operating room. In practice, a simpler approach using a generous amount of medium to high-viscosity ultrasound gel applied directly to the nasal dorsum is likely to provide adequate coupling for axial-plane imaging. Our results should therefore be viewed as supporting the use of a semi-solid standoff pad over a comparable liquid layer in principle, rather than advocating widespread adoption of the specific LGB assembly tested in this study.

The metrics we used are simple ROI-based intensity statistics, but similar approaches—using mean and variance of pixel values and grayscale histograms—have been used to describe B-mode echotexture and contrast differences between normal and diseased tissue in other organs [12,13]. More recent work on ultrasound detectability also emphasizes that the visibility of a structure depends not only on how bright it is but also on how its distribution of intensities compares to that of the background, which is the rationale behind CNR and more advanced generalized CNR measures [15,16]. In that context, higher CNR and SNR under the PAD condition mean that the nasal cortex is more strongly separated from background noise and clutter, which matches what clinicians perceive at the console as “sharper edges” and “cleaner definition” of the fracture line.

The imaging presets in this study (depth 4.0 cm, single focal zone at the cortex) were selected to provide a uniform protocol across a range of nasal sizes and to maintain familiarity for the operators. For even more superficial targets, further reducing the imaging depth and positioning the focal zone slightly closer to the bony interface may increase spatial resolution and edge definition. Systematic evaluation of such optimized presets, in combination with different coupling media, would be a useful direction for future work.

This study has several limitations. First, because of feasibility limitations during the predefined recruitment period, the study included 30 participants. While the primary analyses demonstrated statistically significant differences between conditions, future studies with larger cohorts are warranted to provide more precise effect estimates and to strengthen the generalizability of the results.

Image acquisition and ROI placement were performed in a strictly standardized order by a single operator/reader, measurement variability was minimized, which likely contributed to the high ICC observed in our analyses. However, interobserver variability was not evaluated in this study because all ROI placement and intensity measurements were performed by a single operator and reader. The reproducibility of these ROI-based metrics across different reader cannot be assumed. Image acquisition technique, probe pressure, frame selection, and ROI placement may vary between operators/readers and could influence CNR and SNR estimates. Therefore, our findings should be interpreted in the context of a single operator and reader workflow, and future studies should include several readers and operators to quantify interobserver agreement and to confirm reproducibility.

Although we focused exclusively on quantitative image quality metrics (CNR, SNR_bone, and SNR_soft) and did not systematically evaluate downstream clinical outcomes such as procedure time, reduction success, the need for repeat manipulation, or the requirement for additional imaging, improved near-field image quality with PAD would be expected to facilitate more confident fracture visualization and intraoperative decision-making. It is plausible that such enhancements could translate into better clinical outcomes, but this needs to be confirmed in future studies specifically designed to correlate image quality metrics with patient- and procedure-level endpoints.

## 5. Conclusions

A semi-solid standoff gel pad not only produced higher near-field contrast than a liquid gel barrier but was also straightforward to apply, easy to handle during maneuvers, and readily available as an off-the-shelf consumable; taken together, these attributes make it a clinically useful medium for intraoperative nasal bone sonography. This study provides quantitative evidence supporting the use of a gel pad to optimize real-time ultrasound guidance during closed reduction, while highlighting the need for future work to determine whether improved image quality leads to better clinical outcomes.

## Data Availability

The data presented in this study are available on request from the corresponding author..

## Abbreviations

The following abbreviations are used in this manuscript:

PAD	Standoff gel pad
LGB	Liquid gel barrier
ROI	Region of interest
CNR	Contrast-to-noise ratio
SNR	Signal-to-noise ratio
ICC	Intraclass correlation coefficient
US	High-frequency ultrasonography

## References

[1] Thiede O., Krömer J., Rudack C., Stoll W., Osada N., Schmäl F. (2005). Comparison of Ultrasonography and Conventional Radiography in the Diagnosis of Nasal Fractures. Arch. Otolaryngol. Head Neck Surg..

[2] Hong H.S., Cha J.G., Paik S.H., Park S.J., Park J.S., Kim D.H., Lee H.K. (2007). High-Resolution Sonography for Nasal Fracture in Children. AJR Am. J. Roentgenol..

[3] Park C.H., Yoo H., Yoon K.R. (2009). Usefulness of Ultrasonography in the Treatment of Nasal Bone Fractures. J. Trauma.

[4] Lee M.H., Cha J.G., Hong H.S., Lee J.S., Park S.J., Paik S.H., Lee H.K.H. (2009). Comparison of High-Resolution Ultrasonography and Computed Tomography in the Diagnosis of Nasal Fractures. J. Ultrasound Med..

[5] Astaraki P., Baghchi B., Ahadi M. (2022). Diagnosis of Acute Nasal Fractures Using Ultrasound and CT Scan. Ann. Med. Surg..

[6] Shigemura Y., Ueda K., Akamatsu J., Sugita N., Nuri T., Otsuki Y., Ueda K., Akamatsu J. (2017). Ultrasonographic Images of Nasal Bone Fractures with Water Used as the Coupling Medium. Plast. Reconstr. Surg. Glob. Open.

[7] Balmaseda M.T., Fatehi M.T., Koozekanani S.H., Lee A.L. (1986). Ultrasound Therapy: A Comparative Study of Different Coupling Media. Arch. Phys. Med. Rehabil..

[8] Poltawski L., Watson T. (2007). Relative Transmissivity of Ultrasound Coupling Agents Commonly Used by Therapists in the UK. Ultrasound Med. Biol..

[9] Corvino A., Sandomenico F., Corvino F., Campanino M.R., Verde F., Giurazza F., Tafuri D., Catalano O. (2020). Utility of a Gel Stand-Off Pad in the Detection of Doppler Signal on Focal Nodular Lesions of the Skin. J. Ultrasound.

[10] Zhang Z., Wang H., He S., Zhong Y., Zou H., Cai L., Zhang Y., Wang H. (2023). The Effect of Gel Pads on the Measurement of Superficial Breast Lesions by Shear Wave Elastography. Ann. Med..

[11] Schindelin J., Arganda-Carreras I., Frise E., Kaynig V., Longair M., Pietzsch T., Preibisch S., Rueden C., Saalfeld S., Schmid B. (2012). Fiji: An Open-Source Platform for Biological-Image Analysis. Nat. Methods.

[12] Lee C.H., Kim K.A., Park C.M., Park S.W., Kim B.H., Cha S.H. (2006). Usefulness of Standard Deviation on the Histogram of Ultrasound as a Quantitative Value for Hepatic Parenchymal Echo Texture. Ultrasound Med. Biol..

[13] Ikuta E., Koshiyama M., Watanabe Y., Banba A., Yanagisawa N., Nakagawa M., Ono A., Seki K., Kambe H., Godo T. (2023). A Histogram Analysis of the Pixel Grayscale (Luminous Intensity) of B-Mode Ultrasound Images of the Subcutaneous Layer Predicts the Grade of Leg Edema in Pregnant Women. Healthcare.

[14] Koo T.K., Li M.Y. (2016). A Guideline of Selecting and Reporting Intraclass Correlation Coefficients for Reliability Research. J. Chiropr. Med..

[15] Rodriguez-Molares A., Rindal O.M.H., D’Hooge J., Masoy S.-E., Austeng A., Lediju Bell M.A., Torp H. (2020). The Generalized Contrast-to-Noise Ratio: A Formal Definition for Lesion Detectability. IEEE Trans. Ultrason. Ferroelectr. Freq. Control.

[16] Hyun D., Kim G.B., Bottenus N., Dahl J.J. (2022). Ultrasound Lesion Detectability as a Distance Between Probability Measures. IEEE Trans. Ultrason. Ferroelectr. Freq. Control.

